# Efficacy of Daratumumab-Based Regimens Compared to Standard of Care in Transplant-Eligible Multiple Myeloma: A Meta-Analysis

**DOI:** 10.7759/cureus.15098

**Published:** 2021-05-18

**Authors:** Trishala Menon, Saurabh Kataria, Ramesh Adhikari, Hira Khan, Muhammad Zain Khalid, Mohammad Omar Saeeduddin, Shafaq Taj, Usama Rehman, Aysun Tekin, Romil Singh

**Affiliations:** 1 Family Medicine, Wheeling Hospital, Wheeling, USA; 2 Neurology, Ochsner Louisiana State University Health Sciences Center - Shreveport, Shreveport, USA; 3 Neurology and Neurocritical Care, University of Missouri Health Care, Columbia, USA; 4 Neurology, West Virginia University, Morgantown, USA; 5 Hospital Medicine, Franciscan Health, Lafayette, USA; 6 Geriatrics, Brown University, Providence, USA; 7 Neurology, Allegheny Health Network, Pittsburgh, USA; 8 Internal Medicine, Liaquat National Hospital and Medical College, Karachi, PAK; 9 Psychiatry, Liaquat National Hospital and Medical College, Karachi, PAK; 10 Internal Medicine, Deccan College of Medical Sciences, Hyderabad, IND; 11 Anesthesia, Mayo Hospital, Lahore, PAK; 12 Anesthesia Clinical Research Unit, Mayo Clinic, Rochester, USA; 13 Critical Care, Mayo Clinic, Rochester, USA

**Keywords:** daratumumab, transplant eligible, newly diagnosed, multiple myeloma, immunomodulatory

## Abstract

Daratumumab (dara) belongs to a class of monoclonal antibodies that target CD38 receptors expressed on multiple myeloma (MM) cells. It was first approved for MM treatment in 2015. The efficacy and safety of dara have been reported in many studies. In this analysis, we assessed the outcome of dara addition to standard of care for transplant-eligible newly diagnosed (ND) MM. We conducted a comprehensive search using PubMed, ClinicalTrial.gov, and Embase. Out of 435 articles, we included two randomized clinical trials. We computed the odds ratio (OR) of response rates and risk ratio (RR) of adverse effects using Cochrane RevMan version 5.4. A total of 1,292 patients were enrolled in both trials. The patients were randomized into the control group and the dara group. The dara group included 647 patients and the control group included 645 patients. The CASSIOPEIA trial reported the outcomes using dara, bortezomib (V), thalidomide (T), and dexamethasone (d) versus VTd. The GRIFFIN trial underlined the efficacy of dara, lenalidomide (R), and Vd in the dara group versus RVd in the control group. A pooled analysis of included studies showed an increased overall response rate (OR: 1.60; 95% CI: 1.06-2.41; *p *= 0.02; *I*^2^ = 65%), stringent complete response (OR: 1.59; 95% CI: 1.24-2.05; *p *= 0.03; *I*^2^ = 0%), and negative status for minimal residual disease (OR: 2.47; 95% CI: 1.97-3.10; *p* < 0.01; *I*^2^ = 66%) in the dara group as compared to the control group. However, an increased risk of neutropenia (RR: 1.80; 95% CI: 1.60-2.03; *p* < 0.01) and decreased risk of peripheral neuropathy (RR: 0.92; 95% CI: 0.86-0.99; *p* = 0.02; *I*^2^ = 52%) were observed in the dara group. Dara addition to the standard of care regimen for transplant-eligible NDMM has promising outcomes with increased efficacy and safety profile and manageable toxicity.

## Introduction and background

Multiple myeloma (MM) is a B-cell malignancy characterized by uncontrolled proliferation of plasma cells within the bone marrow with unwarranted monoclonal protein production [1]. MM is an incurable malignancy and the second most common hematological malignancy in the United States and Europe. It accounts for 1.8% of all new cancer cases, 15% of all hematological malignancies, and 2% of all cancer-related deaths in the United States [2]. Initial treatment for newly diagnosed MM (NDMM) depends on the functional status of the patient and whether a patient can tolerate autologous stem cell transplant (ASCT) following the high-dose chemotherapy or not [3]. Young patients without substantial coexisting conditions usually receive an induction regime followed by high dose chemotherapy and ASCT [4].

Daratumumab (dara) belongs to a class of IgGk monoclonal antibodies targeting the CD38 receptors expressed on MM cells and has shown efficacy for newly diagnosed and relapsed or refractory (RR) myeloma as a monotherapy as well as in combination therapy [5-7]. Dara has both direct and indirect antitumor responses, and anti-myeloma activity is based on apoptosis, complement-dependent cytotoxicity, antibody-dependent cellular phagocytosis, and antibody-dependent cell-mediated cytotoxicity [7]. Dara also has an immunomodulatory function that targets and depletes CD38 positive regulator immune suppressor cells [8,9]. It was approved by the U.S. Food and Drug Administration (FDA) in 2015 [10].

The efficacy and safety of dara addition to standard of care therapy were studied in randomized clinical trials in transplant-eligible patients with NDMM. This article has analyzed the effectiveness of dara in phase III/II clinical trials in transplant-eligible patients.

## Review

Material and methods

**Figure 1:**
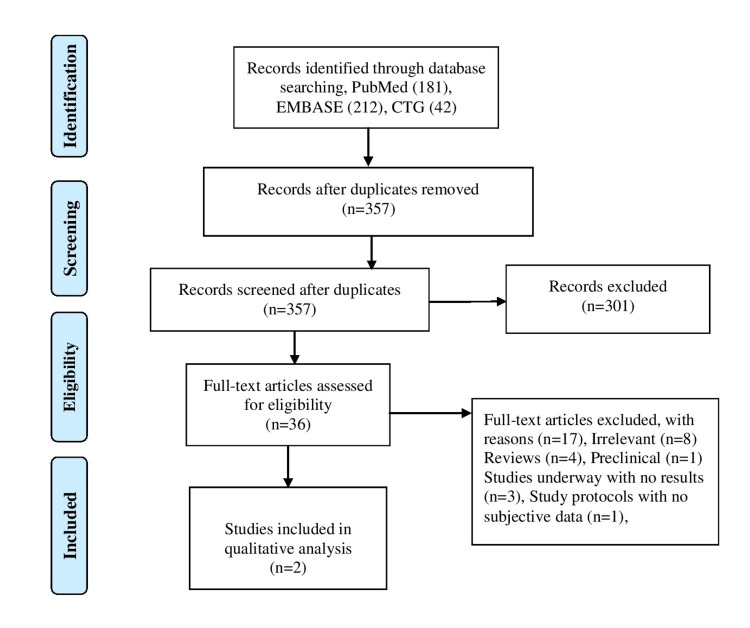
PRISMA flow diagram. PRISMA, Preferred Reporting Items for Systemic Review and Meta-Analyses

We conducted this analysis in accordance with the Preferred Reporting Items for Systemic Review and Meta-analyses (PRISMA) checklist (Figure 1).

Search Strategy and Selection of Studies

We performed a comprehensive literature search using three electronic databases (EMBASE, PubMed, and ClinicalTrials.gov) using the Medical Subject Heading (MeSH) terms and keywords for MM and dara from the date of inception to October 2020. We did not impose any language restrictions. The initial search revealed 435 articles. After excluding the duplicates, review articles, and non-relevant articles, we screened 36 full-length articles. We included two trials in our analysis reporting efficacy and safety of dara-based regimens in randomized clinical trials in transplant-eligible NDMM patients. Two authors (S.T. and H.K.) independently screened the full-length articles for inclusion and exclusion. We imported all the articles to Endnote version 8.0. Two randomized clinical trials met the inclusion criteria. Inclusion criteria included randomized clinical trials reporting the efficacy of dara combined with other regimens compared to standard of care in transplant-eligible NDMM patients.

Study Characteristics and Data Extraction

Two authors (U.R. and A.T.) extracted data from the included clinical trials using a standard excel worksheet. We computed data on study characteristics and outcomes of studies, including overall response rate (ORR), complete response (CR), stringent complete response (sCR), overall survival (OS), progression-free survival (PFS), stable disease (SD), very good partial response (VGPR), progressive disease (PD), and negative status for minimal residual disease (MRD) assessed by multiparametric flow cytometry. Adverse effects (AEs) of the included clinical trials, including hematological toxicity, were also tabulated. Two authors extracted and added the relevant information individually into tables and cross-checked. Any disagreement was resolved through consensus.

Statistical Analysis

We included two randomized clinical trials reporting the efficacy and safety of dara-based regimens in randomized clinical trials in transplant-eligible NDMM patients. We performed a fixed effect analysis to measure the odds ratio (OR) of ORR, MRD, sCR, VGPR, and risk ratio (RR) of hematological and non-hematological toxicities using a 95% confidence interval (CI) in Cochrane RevMan version 5.4. We estimated heterogeneity by the *I*^2^ test, and *I*^2^ statistics were computed in the percentage of the effect size. Values greater than 50% and 70% were considered as moderate and high heterogeneity, respectively.

Results

We included two randomized clinical trials (GRIFFIN and CASSIOPEIA) in our analysis. A total of 1,292 NDMM patients were enrolled in both trials. The patients were randomized into the control group and the dara group. The dara group included 647 patients, and the control group included 645 patients. Voorhees et al. underlined the efficacy in transplant-eligible NDMM patients in the Griffin phase II clinical trial. The regimen included dara, lenalidomide (R), and Vd (bortezomib [V] and dexamethasone [d]) in the dara group versus RVd in the control group [11]. Moreau et al. reported the outcomes of NDMM patients using dara, bortezomib (V), thalidomide (T), and dexamethasone (d) versus VTd in CASSIOPEIA phase III randomized clinical trial [12]. Characteristics of the included studies, including ORR, CR, PFS, OS, and MRD negative status, are shown in Table 1.

**Table 1:** Characteristics of included studies including efficacy profile. ORR, overall response rate; sCR, stringent complete response; PFS, progression-free survival; VGPR, very good partial response; MRD, minimal residual disease; SD, stable disease; PD, progressive disease; Dara, daratumumab; V, bortezomib; R, lenalidomide; T, thalidomide; d, dexamethasone

Parameter	GRIFFIN trial [11]		CASSIOPEIA trial [12]	
	Dara group	Control group	Dara group	Control group
Phase	II		III	
Number of evaluated patients	99	97	543	542
Median age (years)	59	61	59	58
Male	58	60	316	319
Cytogenic standard risk profile	82	83	460	454
Cytogenic high-risk profile	16	14	82	86
Regimen	Dara-VRd	VRd	Dara-VTd	VTd
Median follow-up (months)	22.1	22.1	18.8	18.8
ORR (%)	98	89	90	71
sCR (%)	42	31	29	20
PFS (%)	95.8	89.8	93	85
VGPR (%)	90	71	83	78
Negative status for MRD (%)	51	20.4	64	54
SD (%)	1	7.2	2	3
PD (%)	0	1	4	5

Adverse effects, including hematological and non-hematological toxicities, are computed in Table 2. Responses were measured after a median follow-up of 18.8 months and 22.1 months in the CASSIOPEIA and GRIFFIN trials, respectively.

**Table 2:** Safety profile of the included studies. NA, not available

Adverse effects	GRIFFIN trial [11]				CASSIOPEIA trial [12]			
	Dara group		Control group		Dara group		Control group	
	Any grade	Grade 3/4	Any grade	Grade 3/4	Any grade	Grade 3/4	Any grade	Grade 3/4
Neutropenia (%)	57	41	36	22	29	28	17	15
Thrombocytopenia (%)	43	16	36	09	20	11	14	07
Lymphopenia (%)	30	23	28	22	18	17	12	10
Peripheral neuropathy (%)	59	07	74	08	59	09	63	09
Constipation (%)	51	02	40	01	51	01	49	01
Nausea (%)	49	02	50	01	30	04	24	02
Peripheral edema (%)	34	02	34	03	30	01	28	01
Fusion-related reactions (%)	42	06	NA	NA	35	04	NA	NA

**Figure 2:**
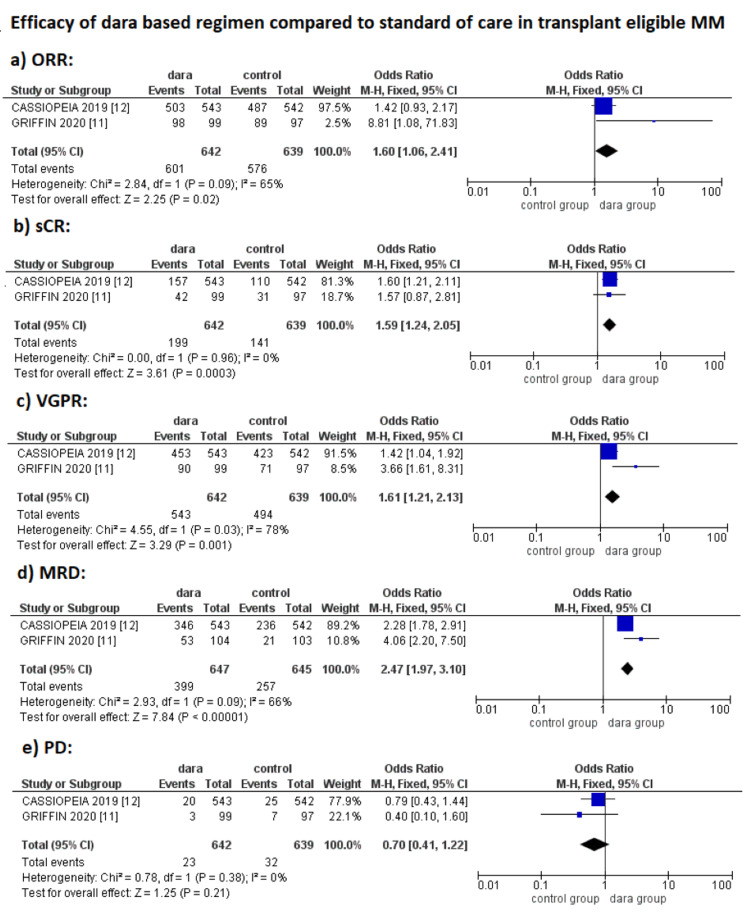
Efficacy profile of daratumumab-based regimens in transplant-eligible NDMM. NDMM, newly diagnosed multiple myeloma; ORR, overall response rate; sCR, stringent complete response; VGPR, very good partial response; MRD, minimal residual disease; PD, progressive disease

A pooled analysis of the included studies showed an increased ORR with an OR of 1.60 (95% CI: 1.06-2.41; p = 0.03: *I*^2^ = 65%), sCR with an OR of 1.59 (95% CI: 1.24-2.05; p = 0.03; *I*^2^ = 0%), and VGPR with an OR of 1.61 (95% CI: 1.21-2.13; p = 0.01; *I*^2^ = 78%) in the dara group as compared to control group. MRD negative status was remarkable in the dara group as compared to the control group, with an OR of 2.47 (95% CI: 1.97-3.10; p < 0.01; *I*^2^ = 66%). A remarkable decrease in PD was also observed in the dara group as compared to the control group, with an OR of 0.70 (95% CI: 0.41-1.22; p < 0.21; *I*^2^ = 0); however, decrease in PD was not significant (Figure 2).

**Figure 3:**
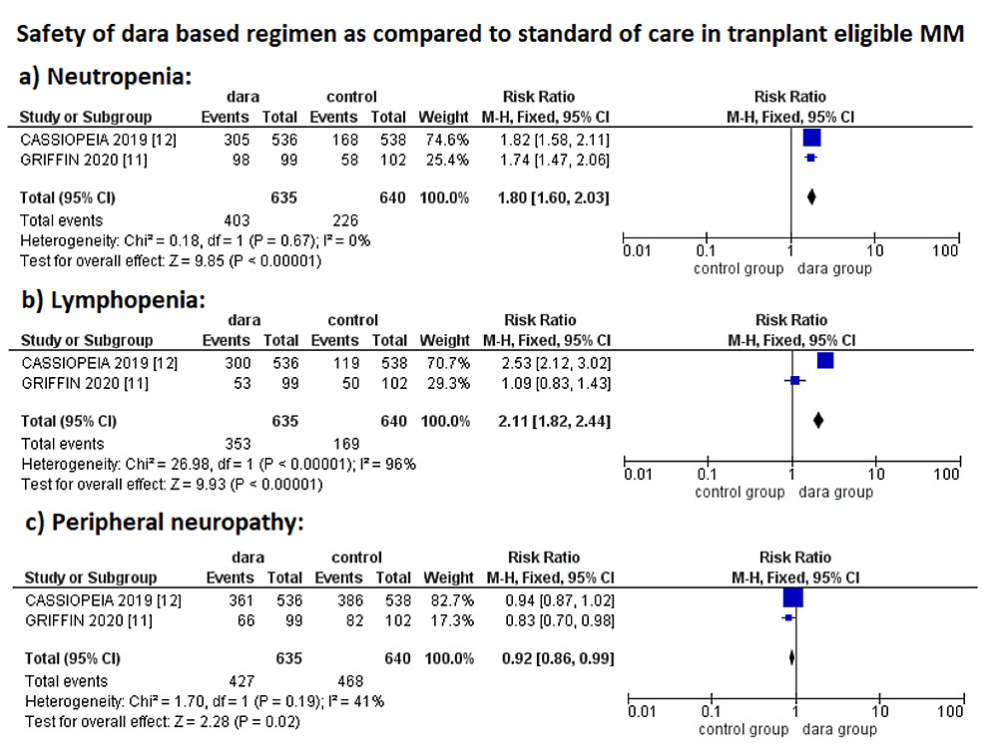
Safety analysis of daratumumab-based regimens in transplant-eligible NDMM. NDMM, newly diagnosed multiple myeloma

However, adverse effects were reported higher in the dara group as compared to the control group, with an increased risk of neutropenia (RR: 1.80; 95% CI: 1.60-2.03; p < 0.01; *I*^2^ = 0%), lymphopenia (RR: 2.11; 95% CI: 1.82-2.44; p < 0.01; *I*^2^ = 96%) and a decreased risk of peripheral neuropathy (RR: 0.92; 95% CI: 0.86-0.99; p = 0.02; *I*^2^ = 41%) (Figure 3).

Discussion

The immunomodulatory drugs (lenalidomide, thalidomide) combined with proteasome inhibitors (bortezomib, carfilzomib) and dexamethasone is the standard of care regimen for transplant-eligible NDMM patients [1]. This frontline therapy has provided extraordinary improvement in long-term patient outcomes; however, the number of patients cured was not many, and long-term treatment led to undesirable toxicities [13,14]. Two randomized clinical trials were conducted to check the efficacy of dara addition to standard of care regimen to reduce the risk of disease progression and increase negative MRD status. The CASSIOPEIA trial conducted the efficacy of dara addition to VTd, and the GRIFFIN trial estimated the efficacy of dara addition to RVd [11,12]. These trials reported better outcomes after the addition of dara with improved depth of response and PFS with acceptable safety (CASSIOPEIA) and lower risk of disease progression or death but more hematological toxicities (GRIFFIN).

Moreau et al. assessed the clinical prognosis of NDMM patients using a dara-based regimen in transplant-eligible NDMM in the CASSIOPEIA trial, and patients were randomized into the dara group (dara-VTd) and the control group (VTd). This study included 1083 patients with a median age of 59 years in the dara group and 58 years in the control group. Patients were given four pre-stem cell transplant inductions and two post-stem cell transplant cycles of both treatment regimens. Out of 543 patients in the dara group, 29% of patients achieved sCR compared to 20% of 542 patients in the VTd group after a follow-up of 18 months. Similarly, a significant reduction in risk of progression or death was seen in the dara group. MRD negative status was also assessed as a secondary endpoint, and it showed marked improvement in the dara group versus the control group. The most common and severe adverse effects were neutropenia, lymphopenia, thrombocytopenia, stomatitis, and pneumonia, as observed in both groups [12]. CASSIOPEIA was the first study to compare the addition of dara to standard therapy in phase III clinical trial. In the Griffin trial, Voorhees et al. assessed the efficacy of using lenalidomide instead of thalidomide. Transplant-eligible patients were randomized into the dara group (dara-VRd) versus the control (VRd) group. This study included 207 patients with a median age of 59 years in the dara group and 61 years in the control group. In the dara group, 42.4% of patients showed sCR, even with a longer follow-up of 22.1 months. Reduction in PD and MRD status improvement was observed in the dara group compared to the control group [11]. Adverse effects mainly were hematological observed in both groups.

Efficacy of dara has also been reported in many studies as a monotherapy alone or in combination with other regimens. Dara has shown efficacy when combined with immunomodulatory drugs and proteasome inhibitors. Landgren et al. conducted a phase II study of carfilzomib (K) plus dara-Rd, and this regimen was continued for eight cycles. Stem cell transplantation was devised after four to six cycles of therapy. Out of 30 enrolled patients, the MRD negativity rate was 75% in 24 patients and ORR was 100%. Phase III trial is underway to evaluate dara-KRd versus KRd [15]. A phase II MASTER trial enrolled 101 patients who received four cycles of dara-KRd as an induction therapy and ASCT and D-KRd as a consolidation therapy. MRD negativity rate was reported among 42% after induction therapy and 82% after consolidation therapy. Side effects mainly were hematological, such as neutropenia, thrombocytopenia, and anemia [16]. Another study assessed the efficacy of dara combined with ixazomib, lenalidomide, and dexamethasone (dara-IxaRd) in MM patients irrespective of the transplant eligibility. In transplant-eligible patients, stem cells were collected after four cycles of dara-IxaRd. Out of 40 enrolled patients, 28% achieved MRD negative status, and VGPR was 69%. Patients tolerated the treatments well, and rash was the most common adverse effect [17].

The efficacy of dara has also been underlined when combined with chemotherapeutic agents and immunomodulatory drugs. A phase II LYRA study enrolled 86 NDMM patients to assess the efficacy of dara in combination with cyclophosphamide, bortezomib, and dexamethasone (dara-CyVd). Overall treatment was safe with VGPR of 44% and ORR of 79% after ASCT with manageable toxicity, including pancytopenia [18]. The same combination was evaluated in phase I study, in which 18 MM patients received dara-CyVd as an induction therapy followed by ASCT. The results were significant in terms of CR (44%), VGPR (94%), and MRD negativity rate (44%) [19].

Our analysis included only two trials reporting outcomes of dara-based regimen than standard care of regimen in transplant-eligible MM. The clinical data in many studies support the enhanced efficacy of dara in combination with proteasome inhibitors and immunomodulatory drugs. Many phase III clinical trials are underway with larger populations to report the outcomes of dara in combination with nuclear export inhibitors (selinexor), proteasome inhibitors (bortezomib), and immunomodulatory drugs (lenalidomide).

## Conclusions

The addition of dara to the standard care regimen for transplant-eligible NDMM has shown effectiveness with favorable toxicity profile, resulting in the widespread clinical use of this monoclonal antibody alone and in combination with standard of care for MM treatment. Growing data from randomized clinical trials are crucial to formulate new treatment combination to improve the surrogate endpoints. Dara showed promising outcomes and favorable efficacy profile in the treatment of transplant-eligible NDMM. Dara also has a possible toxicity profile compared to the control group that is easily manageable by physicians. Given the favorable outcomes of the dara combinations (dara-VTd, dara-VRd) and its manageable toxicity profile, clinical trials to explore other treatment combinations are mandatory.
